# Tablet-Based Cognitive Impairment Screening for Adults With HIV Seeking Clinical Care: Observational Study

**DOI:** 10.2196/25660

**Published:** 2021-09-09

**Authors:** Leah H Rubin, Joan Severson, Thomas D Marcotte, Micah J Savin, Allen Best, Shane Johnson, Joshua Cosman, Michael Merickel, Alison Buchholz, Victor A Del Bene, Lois Eldred, Ned C Sacktor, Joelle-Beverlie Fuchs, Keri N Althoff, Richard D Moore

**Affiliations:** 1 Johns Hopkins University Baltimore, MD United States; 2 Digital Artefacts LLC Iowa City, IA United States; 3 University of California San Diego San Diego, CA United States; 4 Fordham University Bronx, NY United States; 5 Abbvie, Inc Chicago, IL United States; 6 University of Alabama at Birmingham Birmingham, AL United States

**Keywords:** cognitive complications, people with HIV, digital assessment, HIV, tablet, screening

## Abstract

**Background:**

Neurological complications including cognitive impairment persist among people with HIV on antiretrovirals; however, cognitive screening is not routinely conducted in HIV clinics.

**Objective:**

Our objective for this study was 3-fold: (1) to determine the feasibility of implementing an iPad-based cognitive impairment screener among adults seeking HIV care, (2) to examine the psychometric properties of the tool, and (3) to examine predictors of cognitive impairment using the tool.

**Methods:**

A convenience sample of participants completed Brain Baseline Assessment of Cognition and Everyday Functioning (BRACE), which included (1) Trail Making Test Part A, measuring psychomotor speed; (2) Trail Making Test Part B, measuring set-shifting; (3) Stroop Color, measuring processing speed; and (4) the Visual–Spatial Learning Test. Global neuropsychological function was estimated as mean T score performance on the 4 outcomes. Impairment on each test or for the global mean was defined as a T score ≤40. Subgroups of participants repeated the tests 4 weeks or >6 months after completing the first test to evaluate intraperson test–retest reliability and practice effects (improvements in performance due to repeated test exposure). An additional subgroup completed a lengthier cognitive battery concurrently to assess validity. Relevant factors were abstracted from electronic medical records to examine predictors of global neuropsychological function.

**Results:**

The study population consisted of 404 people with HIV (age: mean 53.6 years; race: 332/404, 82% Black; 34/404, 8% White, 10/404, 2% American Indian/Alaskan Native; 28/404, 7% other and 230/404, 58% male; 174/404, 42% female) of whom 99% (402/404) were on antiretroviral therapy. Participants completed BRACE in a mean of 12 minutes (SD 3.2), and impairment was demonstrated by 34% (136/404) on Trail Making Test A, 44% (177/404) on Trail Making Test B, 40% (161/404) on Stroop Color, and 17% (67/404) on Visual-Spatial Learning Test. Global impairment was demonstrated by 103 out of 404 (25%). Test–retest reliability for the subset of participants (n=26) repeating the measure at 4 weeks was 0.81 and for the subset of participants (n=67) repeating the measure almost 1 year later (days: median 294, IQR 50) was 0.63. There were no significant practice effects at either time point (*P*=.20 and *P*=.68, respectively). With respect for validity, the correlation between global impairment on the lengthier cognitive battery and BRACE was 0.63 (n=61; *P*<.001), with 84% sensitivity and 94% specificity to impairment on the lengthier cognitive battery.

**Conclusions:**

We were able to successfully implement BRACE and estimate cognitive impairment burden in the context of routine clinic care. BRACE was also shown to have good psychometric properties. This easy-to-use tool in clinical settings may facilitate the care needs of people with HIV as cognitive impairment continues to remain a concern in people with HIV.

## Introduction

Thirty-six years into the HIV epidemic, North America has had markedly improved clinical outcomes and prolonged life. Death from non-AIDS comorbidities is now more common than AIDS-related death, and life-expectancy has increased markedly among those on antiretroviral therapy [1-5]. Similarly, AIDS-related comorbidities are now less common than noncommunicable, age-related comorbidities. Cognitive impairment among people with HIV persists despite effective antiretroviral therapy [6-9]. Cognitive impairment in the current treatment era is often mild and not readily detectable to the practicing clinician. At present, only clinical criteria and neuropsychological testing are used to diagnose cognitive impairment, and no single laboratory test or biomarker has been established to effectively detect mild cognitive impairment. Current screening measures (eg, the International HIV Dementia Scale [10], Montreal Cognitive Assessment [11], HIV Dementia Scale [12]) lack sensitivity for detecting milder forms of cognitive impairment [13-18], and the resources (eg, time, cost, training) required for comprehensive neuropsychological assessments limits their widespread use during routine clinic visits. Thus, there is a pressing need for brief screening measures that could be easily implemented into routine clinic care in order to determine persons in need of comprehensive neuropsychological evaluation.

Tablet computing tools, such as the Apple iPad, are increasingly ubiquitous and offer an opportunity to potentially implement an intuitive interface for primarily self-directed brief cognitive assessments with automated scoring, data aggregation, and preliminary screening of impairment in real time, thus minimizing clinician and staff burden and increasing the opportunity to identify individuals in need of neuropsychological evaluation. Recently, a brief iPad tool was developed to screen cognitive impairment. The testing platform has automated data aggregation, which provides global data to facilitate and or support computational epidemiological applications, pharmaceutical development, and clinical trial monitoring, including monitoring of the effectiveness of antiretroviral therapy and nonantiretroviral therapy medications on cognitive impairment in the context of clinical care. The largely self-administered testing, survey, and automated reporting has the potential to be used as a model for the design and development of mobile app to quantify cognition, behavior, mental health, and mobility in the clinic and through emerging mobile technologies worldwide.

Herein we first aimed to determine the feasibility of using an iPad-based tool (BRACE, Brain Baseline Assessment of Cognition and Everyday Functioning) to screen for cognitive impairment among adults with HIV seeking clinical care in Baltimore, Maryland. Second, we aimed to examine the psychometric properties of the iPad-based cognitive screener including test–retest reliability and practice effects (improvement in performance from repeated exposures to testing materials) as well as validity. Third, we aimed to understand predictors (sociodemographic, clinical, and behavioral data) of cognitive impairment using the iPad-based tool among people with HIV.

## Methods

### Study Population

From January 29, 2019 to December 30, 2019, a convenience sample of patients was recruited during routine clinic visits (via the clinic’s research desk or by provider referral) in the John G. Bartlett HIV Practice at the Johns Hopkins Hospital in Baltimore, Maryland. Inclusion criteria were minimal and only included (1) English-language proficiency and (2) being able to provide informed consent. There were no postconsent exclusion criteria because our goal was to determine feasibility of integrating BRACE in the context of routine clinic care for people with HIV rather than focus on HIV-associated neurocognitive disorders, which are only deemed present if the cognitive impairment cannot be attributed to any other comorbid condition or other confounders [19]. This study was conducted in accordance with ethical standards for human experimentation and was approved by the Johns Hopkins School of Medicine Institutional Review Board.

### Procedure

Staff at the clinic’s research desk and providers directed interested patients to trained study research assistants. After confirming that patients met initial eligibility criteria, research assistants explained the study and obtained informed consent. Patients who enrolled completed the BRACE and the Computerized Adaptive Test for Mental Health. All visits were conducted in exam rooms with a research coordinator to help with administration (no staff monitoring). After completing the study visit, participants were added to our Clinical Research Management System to ensure that each patient only consented once to the study. Clinical Research Management System data entry was also important to identify when patients were due for their routine clinic visits. Due to the nature of the study, there was more flexibility in administration of follow-up visits (eg, follow-up completion of the BRACE occurred when participants’ clinic visits fell on or after the 6-month follow-up mark). This study only relied on performance on the BRACE at these visits. A subset of patients also completed a lengthier cognitive test battery as part of other ongoing neuroHIV clinical studies the same day as the completion of BRACE. The order of administration (BRACE before or after the lengthier battery) was based on patients’ schedules; we typically schedule our neuroHIV studies on the same day as clinic appointments.

### Cognitive Function Outcomes

This self-administered tool (automated audio and video instructions) used 4 validated neuropsychological tests.

The BRACE tool includes the Trail Making Test (TMT) Part A, which measures psychomotor speed, TMT Part B, which measures set-shifting and mental flexibility, Stroop Color Test, which measures processing speed, and Visual-Spatial Learning Test, which measures visuospatial learning and memory. BRACE has been shown to have high sensitivity to HIV-related or other brain dysfunction; during its development, *T* scores (mean 50, SD 10) were generated using a normative based regression approach (adjusted for age, sex, race/ethnicity, and education) based on a sample of 144 HIV-uninfected, healthy individuals free from significant confounds that might affect cognitive performance (eg, recent or significant traumatic brain injury, neurologic disorder, central nervous system infections, etc). The normative group was a mean age of 42.2 years (SD 15.7, range 18-70), with education level of 15.2 years (SD 2.26, range 9-20) with 45.8% being male and 56.9% White (T Marcotte, unpublished data). Six-month test–retest reliability (n=110) for the overall score was *r*=0.84. In an independent validation (T Marcotte, unpublished data) with 109 participants (66 people with HIV, 43 HIV-uninfected), a significant difference (*P*<.001) and a large effect size of 1.18 between the people with HIV and HIV-uninfected were found; the HIV-uninfected group had a mean *T* score of 50.8, suggesting the norms worked well when applied to this additional group.

A global neuropsychological score was computed by averaging performance across the 4 outcomes [9]. Impairment was defined as *T* score <40, based upon maximizing sensitivity and specificity relative to the full neuropsychological battery. The tool also includes an abbreviated version of the Patient’s Assessment of Own Functioning Inventory, a measure of self-reported cognitive complaints consisting of 5 dimensions, and the Patient Health Questionnaire–2, a measure inquiring about the frequency of depressed mood and anhedonia over the past 2 weeks. Significant self-reported cognitive symptoms were determined based upon regression-based analyses of the full Patient’s Assessment of Own Functioning Inventory. A score of 3 or greater on the Patient Health Questionnaire–2 was considered being at-risk for depression [20].

We retested BRACE in a subset of individuals within 30 days of initial testing (n=26) and more than 6 months after initial testing (n=67).

### Neuropsychological Test Battery

Within our study population, a subset of 61 participants also completed a lengthier cognitive test battery as part of other ongoing neuroHIV clinical studies on the same day that they completed BRACE. This subset was similar to rest of the study population on most factors except for sex, with the subsample having more women than the larger group had (Table S1 in Multimedia Appendix 1). The neuropsychological test battery included the following tests: (1) Hopkins Verbal Learning Test revised, which measures auditory-verbal learning and memory, (2) TMT Parts A and B, (3) Grooved Pegboard Test, which measures fine motor speed and dexterity, (4) Digit Symbol Modalities Test, which measures processing speed, and (5) Animal Fluency, which measures semantic verbal fluency. The completion order of the neuropsychological test battery and BRACE was not systematically assigned or tracked; some participants went from a routine clinical visit to a neuroHIV clinical study visit or vice versa. All outcome measures from these tests were standardized using regression-based equations from HIV-uninfected individuals participating in the Women’s Interagency HIV Study and the Multicenter AIDS Cohort Study [21,22]. Thus, all outcomes were in *z* score units (mean 0, SD 1). A global neuropsychological function score was computed by averaging all outcome measures. Impairment was defined a priori as performing 1 SD below the global neuropsychological mean [9].

### Covariates: Demographic Characteristics, HIV Biomarkers, Antiretroviral Therapy Medication, and Comorbidities

Patient-level variables were extracted and validated by 2 research coordinators. Sociodemographic factors included age, sex, race/ethnicity, and years of education. HIV-related clinical factors included the closest (until or on the day of assessment) plasma CD4 count and HIV RNA (lower limit of detection: 20 copies per mL) in the electronic medical record, and antiretroviral therapy medications (name and class of medication).

Additionally, we focused on extracting medical comorbidities within 4 general International Statistical Classification of Diseases and Related Health Problems (ICD-10) categories in the electronic medical record: (1) endocrine, nutritional, and metabolic diseases (ICD-10 codes E00-E99); (2) mental, behavioral, and neurodevelopmental disorders including substance use disorders (ICD-10 codes F01-F99); (3) nervous system disorders (ICD-10 codes G00-G99); and (4) circulatory system issues (ICD-10 codes I00-I99). These problems were selected as our focus as these comorbidities have known associations with cognitive health in HIV [23-26].

### Statistical Analysis

Descriptive statistics were used to characterize the study population and the prevalence of cognitive impairment. Pearson correlations were used to examine the association between performance on the BRACE initially and at a subsequent time point. Practice effects (performance initially vs a subsequent time point) were examined using a paired-sample *t* test. Pearson correlations were also used to examine the association between performance on BRACE and performance on the lengthier neuropsychological test battery. Logistic regression models were used to explore the univariable and multivariable associations of the covariates with the outcomes. Covariates included in the multivariable logistic regression models included those with a statistically significant univariable association and demographic characteristics with face validity, including age, sex, race/ethnicity, and HIV acquisition risk group. All analyses were conducted in SAS software (version 9.4; SAS Institute Inc), and a *P* value <.05 indicated statistical significance.

## Results

### Participant Characteristics

The study population included 404 people with HIV (Table 1; age: range 21.6 to 79.3 years). Of the population, 99.5% (402/404) were currently on antiretroviral therapy, and 66.1% (267/404) had an undetectable viral load (<20 copies per mL) near the time of cognitive impairment assessment (median 10 days, IQR 63). The median CD4 level was 631 cells/μL (IQR 476) near the time of cognitive assessment (median 31 days, IQR 63). The most commonly prescribed antiretroviral therapy agents included nucleoside reverse-transcriptase inhibitors emtricitabine (258/404, 63.9%) and tenofovir alafenamide (245/404, 60.6%), protease inhibitor darunavir (94/404, 23.3%), and integrase inhibitors dolutegravir (170/404, 42.1%) and bictegravir (92/404, 22.8%).

Table 2 provides the most common (>5%) ICD-10 problems listed under 4 categories of comorbidities (endocrine, nutritional, and metabolic diseases; mental, behavioral, and neurodevelopmental disorders and substance use disorders; nervous system disorders; circulatory system issues).

On the iPad, 18.1% (73/404) had Patient Health Questionnaire-2 scores suggesting possible risk for depression, and 66.1% (267/404) perceived significant impairments in daily activities.

**Table 1:** Sociodemographic, behavioral, and clinical characteristics in the overall sample of people with HIV seeking clinical care and by cognitive impairment status based upon Brain Baseline Assessment of Cognition and Everyday Functioning performance.

Characteristic		Overall (N=404)	Impaired (n=103)	Not impaired (n=301)	*P* value
Age, mean (SD)		53.6 (10.7)	50.2 (10.5)	54.8 (10.5)	<.001
Age >50 years, n (%)		290 (71.8)	64 (62.1)	226 (75.1)	.01
Age >60 years, n (%)		123 (30.4)	13 (12.6)	110 (36.5)	<.001
Male, n (%)		230 (56.9)	63 (61.2)	167 (55.5)	.31
**Education^a^, n (%)**					.97
	Less than high school	119 (29.5)	30 (29.1)	89 (29.6)	
	High school	172 (42.6)	44 (42.7)	128 (42.5)	
	More than high school	111 (27.5)	27 (26.2)	84 (27.9)	
**Race, n (%)**					.36
	African-American/Black	332 (82.2)	81 (78.6)	251 (83.4)	
	Caucasian/White	34 (8.4)	8 (7.8)	26 (8.6)	
	American Indian/Alaskan Native	10 (2.5)	3 (2.9)	7 (2.3)	
	Other	28 (6.9)	11 (10.7)	17 (5.6)	
Hispanic/Latino ethnicity, n (%)		12 (3.0)	7 (6.8)	5 (1.7)	.008
**Current CD4 count^b^, n (%)**					.24
	Less than 200	43 (10.6)	15 (14.6)	28 (9.3)	
	200-500	91 (22.5)	24 (23.3)	67 (22.3)	
	More than 500	264 (65.3)	61 (59.2)	203 (67.4)	
**Current HIV RNA (copies per milliliter)^b^, n (%)**					.43
	Undetectable (<20)	266 (65.8)	62 (60.2)	204 (67.8)	
	Less than 200	83 (20.5)	26 (25.2)	57 (18.9)	
	Greater than 200	49 (12.1)	12 (11.7)	37 (12.3)	
On antiretroviral therapy, n (%)		402 (99.5)	103 (100)	299 (99.3)	.41
On antiretroviral therapy and undetectable HIV RNA, n (%)		265 (65.6)	62 (60.2)	203 (67.4)	.18
**Nucleoside reverse-transcriptase inhibitor, n (%)**					
	Emtricitabine	258 (63.9)	67 (65.0)	191 (63.5)	.77
	Tenofovir alafenamide	245 (60.6)	64 (62.1)	181 (60.1)	.72
	Abacavir	82 (20.3)	21 (20.4)	61 (20.3)	.98
	Lamivudine	77 (19.1)	19 (18.4)	58 (19.3)	.85
**Nonnucleoside reverse-transcriptase inhibitor, n (%)**					
	Rilpivirine	39 (9.7)	8 (7.8)	31 (10.3)	.45
**Protease inhibitor, n (%)**					
	Darunavir	94 (23.3)	25 (24.3)	69 (22.9)	.78
	Ritonavir	44 (10.9)	10 (9.7)	34 (11.3)	.65
**Integrase inhibitor, n (%)**					
	Dolutegravir	170 (42.1)	46 (44.7)	124 (41.2)	.54
	Bictegravir	92 (22.8)	25 (24.3)	67 (22.3)	.67
	Elvitegravir	52 (12.9)	11 (10.7)	41 (13.6)	.44
	Raltegravir	21 (5.2)	4 (3.9)	17 (5.6)	.49

^a^Data are missing from 2 participants.

^b^Data are missing from 4 participants; antiretroviral therapy included are agents used by more than 5% of the sample.

**Table 2:** Common ICD-10 codes from medical records in the overall sample of people with HIV seeking clinical care and by Brain Baseline Assessment of Cognition and Everyday Functioning cognitive impairment status.

ICD-10^a^		Overall (N=404), N (%)	Impaired (n=103), n (%)	Normal (n=301), n (%)	*P* value
**Endocrine, nutritional, and metabolic diseases (ICD-10 E00-E89)**		239 (59.2)	57 (55.3)	182 (60.5)	.36
	Metabolic disorders (ICD-10 E70-E88)	144 (35.6)	32 (31.1)	112 (37.2)	.26
	Overweight, obesity and other hyperalimentation (ICD-10 E65-E68)	73 (18.1)	14 (13.6)	59 (19.6)	.17
	Diabetes (ICD-10 E8-E13)	71 (17.6)	17 (16.5)	54 (17.9)	.74
	Disorders of other endocrine glands (ICD-10 E20-E35)	30 (7.4)	5 (4.9)	25 (8.3)	.25
**Mental, behavioral, and neurodevelopmental disorders (ICD-10 F00-F99)**		330 (81.7)	83 (80.6)	247 (82.1)	.74
	Mood [affective] disorders (ICD-10 F30-F39)	234 (57.9)	58 (56.3)	176 (58.5)	.70
	Mental and behavioral disorders due to psychoactive substance use (F10-F19)	222 (55.0)	53 (51.5)	169 (56.1)	.41
	Other psychoactive substance related disorders (ICD-10 F19)	117 (29.0)	31 (30.1)	86 (28.6)	.77
	Nicotine dependence (ICD-10 F17)	84 (20.8)	18 (17.5)	66 (21.9)	.34
	Alcohol related disorders (ICD-10 F10)	61 (15.1)	16 (15.5)	45 (15.0)	.88
	Opioid (ICD-10 F11)	63 (15.6)	19 (18.4)	44 (14.6)	.35
	Cocaine (ICD-10 F14)	59 (14.6)	15 (14.6)	44 (14.6)	.99
	Cannabis (ICD-10 F12)	15 (3.7)	2 (1.9)	13 (4.3)	.27
	Anxiety, dissociative, stress-related, somatoform (ICD-10 F40-F48)	64 (15.8)	11 (10.7)	53 (17.6)	.10
	Psychosis (ICD-10 F20-F29)	13 (3.2)	5 (4.9)	8 (2.7)	.28
**Diseases of the nervous system (ICD-10 G00-G99)**		163 (40.3)	43 (41.7)	120 (39.9)	.74
	Episodic and paroxysmal disorders (ICD-10 G40-G47)	82 (20.3)	22 (21.4)	60 (19.9)	.76
	Polyneuropathies and other disorders of the PNS (ICD-10 G60-G65)	66 (16.3)	13 (12.6)	53 (17.6)	.24
	Nerve, nerve root and plexus disorder (ICD-10 G50-G59)	29 (7.2)	7 (6.8)	22 (7.3)	.86
**Diseases of the circulatory system (ICD-10 I00-I99)**		244 (60.4)	54 (52.4)	190 (63.1)	.06
	Hypertensive diseases (ICD-10 I10-I16)	192 (47.5)	41 (39.8)	151 (50.2)	.07
	Ischemic heart disease (ICD-10 I20-I25)	64 (15.8)	7 (6.8)	57 (18.9)	.004
	Other forms of heart disease (ICD-10 I30-I55)	51 (12.6)	6 (5.8)	26 (8.6)	.36
	Diseases of veins, lymphatic vessels and lymph nodes (ICD-10 I80-I89)	40 (9.9)	8 (7.8)	32 (10.6)	.40
	Pulmonary heart disease and disease of pulmonary circulation (ICD-10 I26-I28)	33 (8.2)	5 (4.9)	28 (9.3)	.15
	Disease of arteries, arterioles and capillaries (ICD-10 I70-I79)	32 (7.9)	6 (5.8)	26 (8.6)	.36
	Cerebrovascular disease (ICD-10 I60-I69)	25 (6.2)	9 (8.7)	16 (5.3)	.21

^a^ICD-10 International Statistical Classification of Diseases, tenth revision.

### Cognitive Function in People With HIV Seeking Clinical Care

**Figure 1:**
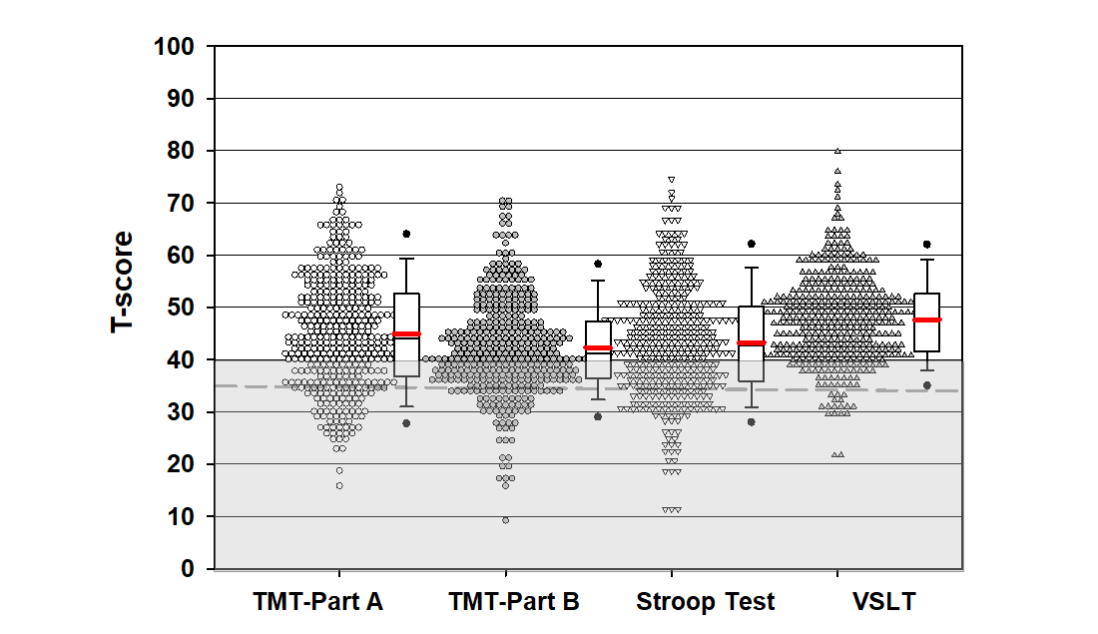
Performance on iPad cognitive assessment tool of people with HIV seeking clinical care. The red line indicates the mean, the grey shaded section indicates the score is in the range of impairment (T score <40); the dotted grey line is T score=35 (1.5 SD below the mean). TMT: Trail Making Test; VSLT: Visual Spatial Learning Test.

**Figure 2:**
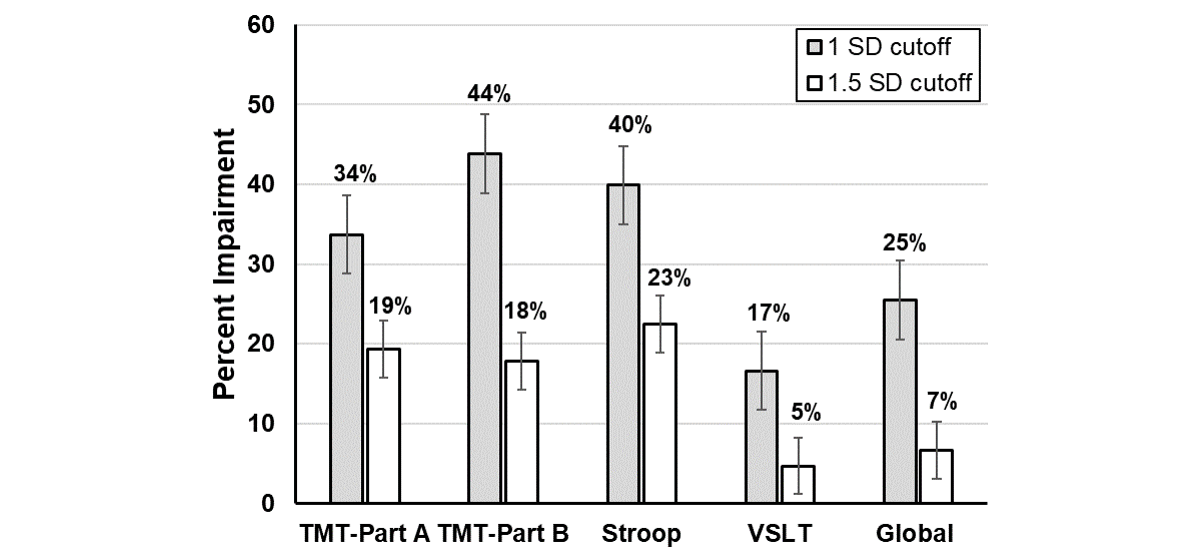
Percentage impairment in study population of people with HIV seeking clinical care. TMT: Trail Making Test; VSLT: Visual Spatial Learning Test.

The mean completion time for BRACE for the older individuals in the study population was 12 minutes (SD 3.2). The average *T* score on TMT A was 44.9 (SD 10.7), TMT B was 42.4 (SD 9.3), Stroop was 43.2 (SD 10.5), Visual-Spatial Learning Test was 47.7 (SD 8.4), and global neuropsychological function was 44.6 (SD 7.2) (Figure 1 and Figure 2; Tables S2 and S3 in Multimedia Appendix 1). When using the definition of impairment of 1 SD below the mean (*T* score <40), 33.7% (136/404) were impaired on TMT A, 43.8% (177/404) on TMT B, 39.9% (161/404) on Stroop, 16.6% (67/404) on Visual-Spatial Learning Test, and 25.5% (103/404) on global neuropsychological function.

**Figure 3:**
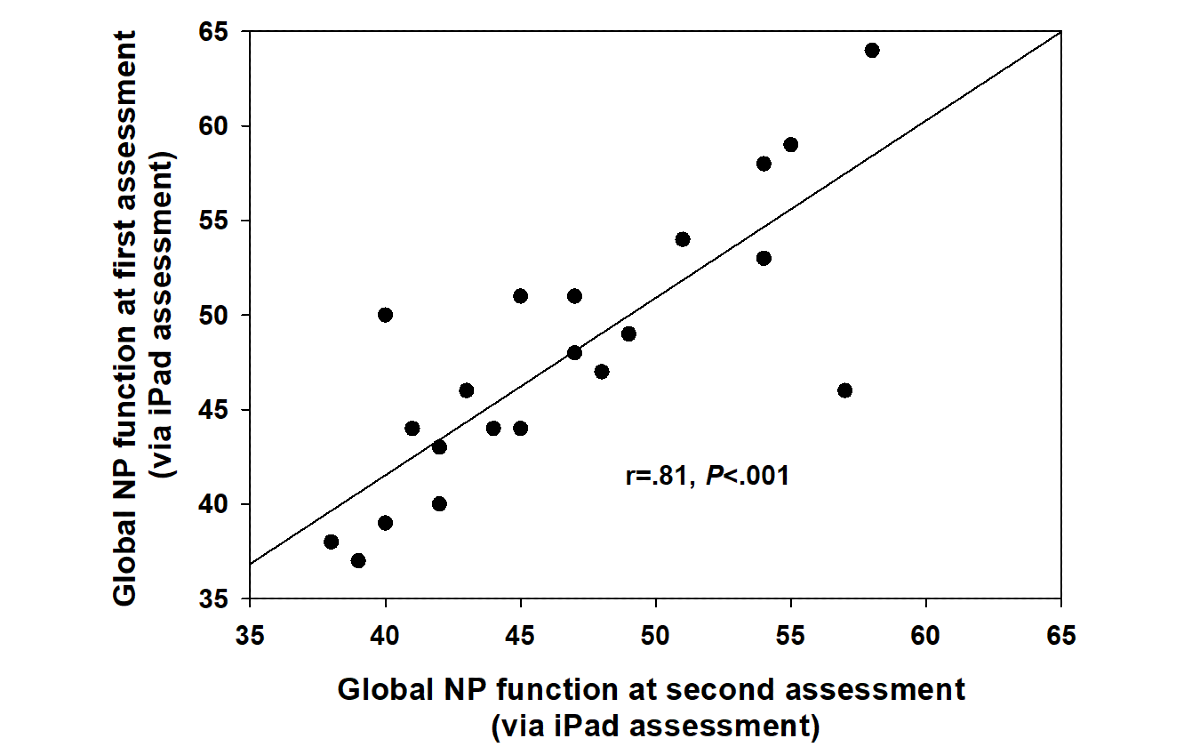
Associations between global neuropsychological function assessed with the tool at the initial time point and 30 days later in 26 people with HIV assessed via the gold standard neuropsychological battery in 61 people with HIV.

**Figure 4:**
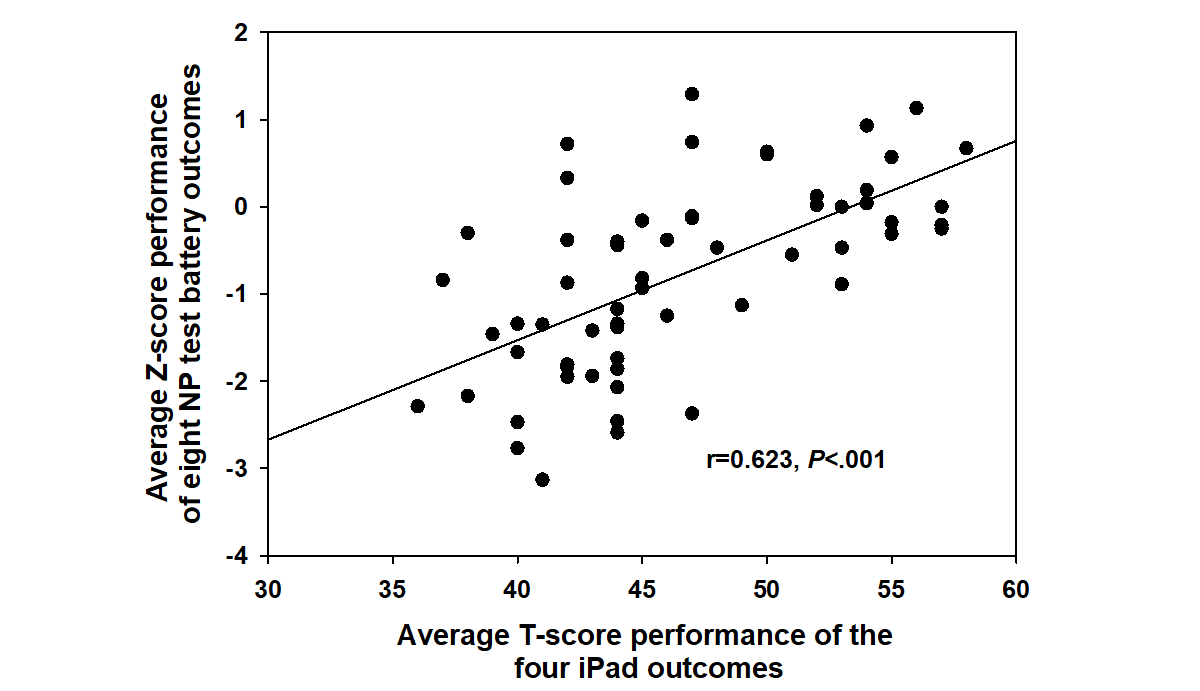
Associations between global neuropsychological function assessed with the tool at the initial time point and almost 1 year later in 67 people with HIV assessed via the gold standard neuropsychological battery in 61 people with HIV.

Of the 404 participants, 26 completed BRACE 30 days later. Test–retest reliability for the subset of participants repeating the measure was 0.81 (Figure 3). There were no significant practice effects (*P*=.20) as the global neuropsychological mean at the first assessment was 46.6 (SD 5.8) and that at the second assessment was 47.72 (SD 6.7). Of the 404 participants, 67 (16.6%) completed BRACE more than 6 months later (days: median 294 days, IQR 50). Test–retest reliability for the subset of participants repeating BRACE almost 1 year later was 0.63 (Figure 4). There were no significant practice effects (*P*=.68) as the global neuropsychological mean at the first assessment was 43.9 (SD 6.2) and the second assessment was 44.1 (SD 5.9).

**Figure 5:**
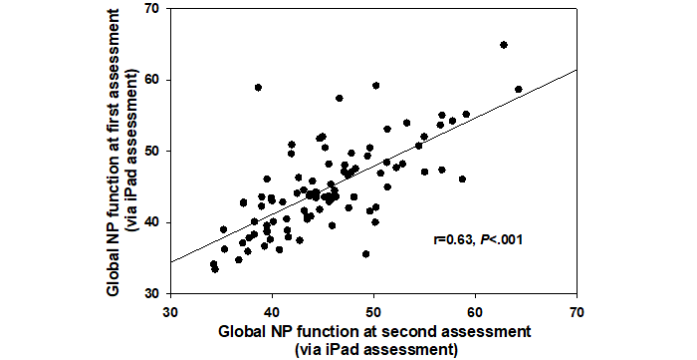
Associations between global neuropsychological function assessed with the tool at the initial time point and global neuropsychological function assessed via the gold standard neuropsychological battery in 61 people with HIV.

**Figure 6:**
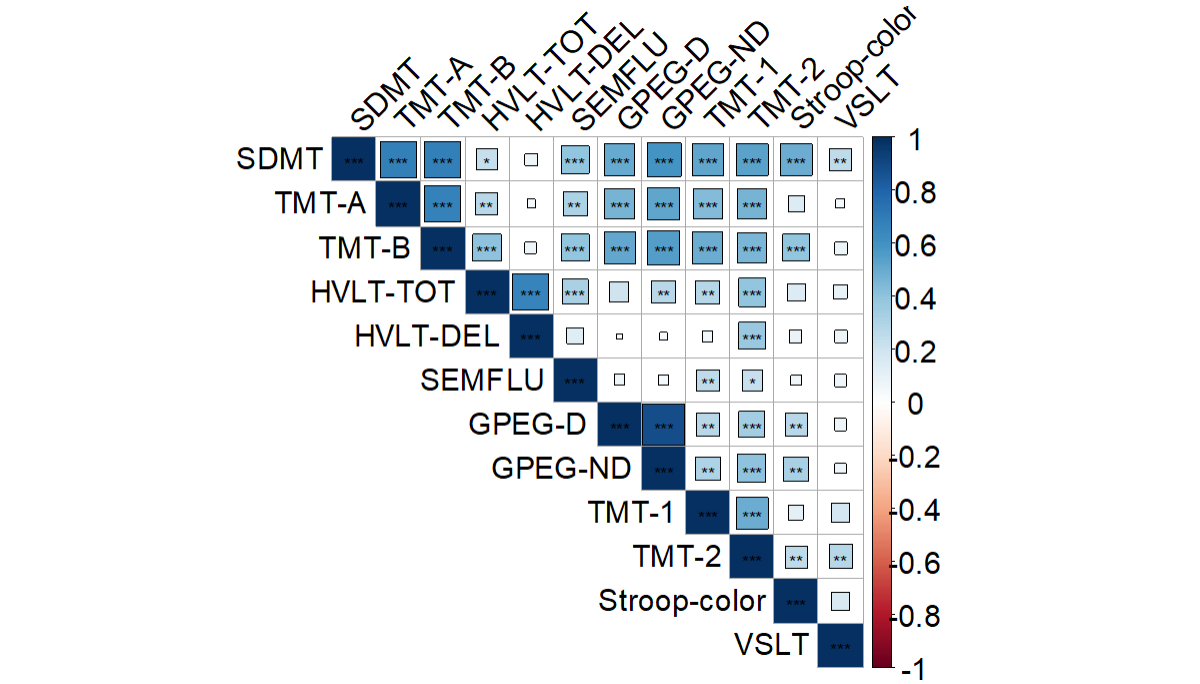
Correlation heatmap between the individual outcomes assessed with the tool and the gold standard neuropsychological test battery. ***P<.001; **P<.01; *P<.05. SDMT: Symbol Digit Modalities Test; DMT: Symbol Digit Modalities Test; TMT: Trial Making Test; HVLT: Hopkins Verbal Learning Test-Revised; SEMFLU: semantic fluency; GPEG-D: Grooved Pegboard dominant hand; GPEG-ND: Grooved Pegboard nondominant hand; VSLT:Visual Spatial Learning Test.

**Figure 7:**
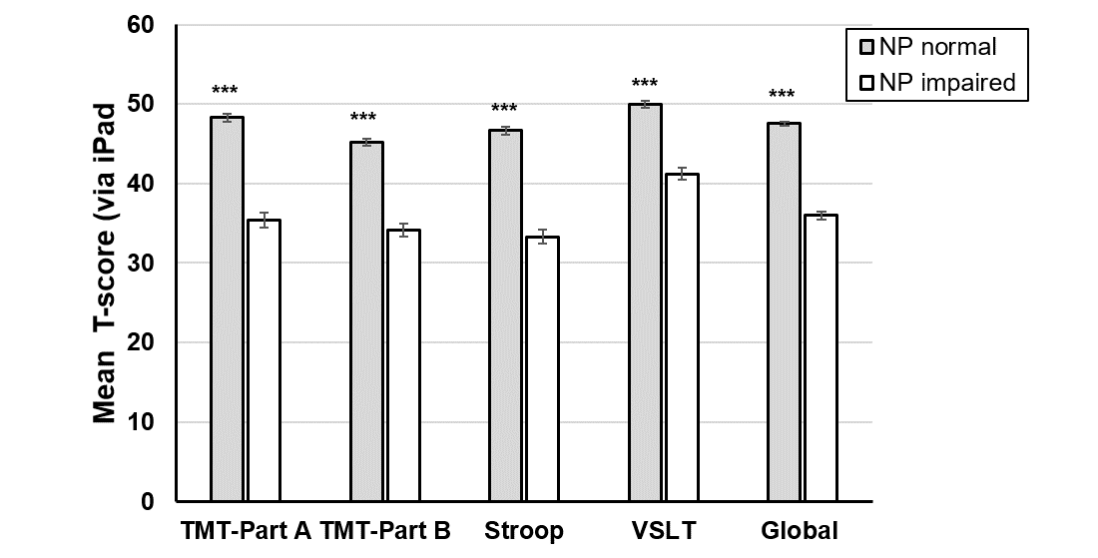
Performance (T scores) on the Brain Baseline Assessment of Cognition and Everyday Functioning iPad cognitive assessment as a function of global neuropsychological impairment (normal vs impaired based on a z score <1) using the gold standard neuropsychological test battery in people with HIV. ***P<.001. TMT: Trail Making Test; VSLT: Visual Spatial Learning Test.

The correlation between the lengthier cognitive test battery and global neuropsychological function via the iPad-based assessment in the subgroup of 61 at the first visit was 0.634 (*P*<.001; Figure 5). This subgroup comprised significantly fewer males (14/61, 23.0%) than the larger sample (216/343, 63.0%, *P*<.001). When examining the degree to which the BRACE outcomes were correlated with the lengthier cognitive test battery outcomes, all associations were in the expected direction, with higher performance on BRACE outcomes correlated with higher performance on neuropsychological test battery outcomes (Figure 6). BRACE also demonstrated good discriminant validity when differentiating between people with HIV with and without global neuropsychological impairment (using a *T* score cutoff of 40) on the gold standard neuropsychological test battery (Figure 7). Using a *T* score cutoff of 40 for global neuropsychological function on BRACE yielded 0.84 sensitivity and 0.94 specificity when compared to global neuropsychological impairment using gold standard neuropsychological tests.

### Predictors

People with HIV demonstrating global neuropsychological impairment using the 1 SD cutoff on the BRACE screen were similar to cognitively healthy people with HIV on the majority of sociodemographic, clinical, and behavioral characteristics (Tables 1, 2, and 3). However, individuals demonstrating global neuropsychological impairment were younger (*P*<.001), more likely to be Hispanic/Latino (*P*=.008), and less likely to have ischemic heart disease (*P*=.004). In a multivariable logistic regression model, both age (*P*=.03) and Hispanic/Latino ethnicity (*P*=.02) were the only significant predictors of global neuropsychological impairment.

**Table 3:** Unadjusted and adjusted odds of cognitive impairment (1 SD cutoff) on Brain Baseline Assessment of Cognition and Everyday Functioning for sociodemographic, clinical, and behavioral factors in the overall sample of people with HIV seeking clinical care.

Factors			Univariable analyses, OR^a^ (95% CI)		Multivariable analysis, OR (95% CI)
Greater than or equal to 50 years of age (vs less than 50 years of age)			0.54 (0.34-0.88)^*^		0.56 (0.33-0.96)^*^
Female (vs male)			1.26 (0.80-1.99)		1.27 (0.76-2.12)
Less than high school (vs high school or more)			0.98 (0.60-1.60)		1.07 (0.62-1.85)
African-American/Black			1.36 (0.78-2.39)		1.03 (0.55-1.93)
Hispanic/Latino			4.32 (1.34-13.91)^**^		4.31 (1.25-14.90)^*^
Current CD4 count fewer than 200 (vs > 200)			1.72 (0.88-3.37)		1.65 (0.80-3.40)
On antiretroviral therapy+ undetectable current HIV RNA (vs antiretroviral therapy+ detectable HIV RNA^b^)			0.71 (.45-1.12)		0.86 (0.51-1.44)
**ICD-10 codes (any vs none)**					
	Endocrine, nutritional, and metabolic diseases	0.81 (0.52-1.27)		1.04 (0.62-1.74)	
	Mental, behavioral, and neurodevelopmental disorders	0.91 (0.51-1.61)		0.90 (0.49-1.65)	
	Diseases of the nervous system	1.08 (0.69-1.70)		1.10 (0.67-1.80)	
	Diseases of the circulatory system	0.64 (0.41-1.01)		0.87 (0.51-1.48)	

^a^OR: odds ratio.

^b^2 cases were not on antiretroviral therapy and were undetectable.

^*^P<.05.

^**^P<.01.

## Discussion

BRACE was developed to briefly screen for cognitive impairment, particularly mild impairment that is not readily detectable by the practicing clinician. In our sample of 404 adults with HIV seeking outpatient clinical care, we demonstrated that this brief, self-administered screener of cognitive impairment can be self-administered rather rapidly in clinic (approximately 7-10 minutes; slightly longer in older adults, approximately 12 minutes) during routine clinic visits. Important to note is that our sample comprised predominately older, African-American/Black individuals with low education and a high burden of mental and behavioral health disorders, hypertension, and metabolic disorders based on electronic medical record. Thus, one of the strengths of the tool is that it can be used in persons who are nonreaders or with low literacy; the tests are not literacy dependent as both written and verbal instructions (via video) are provided. Importantly, the tool has excellent test–retest reliability, no practice effects over a 30-day or a median of approximately 10 months, strongly correlates to a briefer cognitive test battery, and has good classification accuracy compared to the lengthier cognitive test battery, which required 20 to 30 minutes to complete.

The global burden of cognitive impairment in this population using the standard 1 SD cutpoint on BRACE was 25% (103/404) with varying estimates of impairment across each test (67/404, 16.6% to 177/404, 43.8%). Estimates of global neuropsychological impairment using BRACE are consistent with those in previous studies—7% to 60% of people with HIV demonstrated cognitive impairment via neuropsychological testing [6-9,27,28] or other tablet-based tools to assess cognitive impairment, such as NeuroScreen [29].

In addition to estimating the burden of cognitive impairment, our large sample size enabled us to also examine covariates and risk factors for global neuropsychological impairment. Relative to the number of factors extracted from medical records, very few of these factors were associated with global neuropsychological impairment. Some of the factors that emerged are well-established sociodemographic factors including age and ethnicity [30]. Age emerged as a significant predictor of global neuropsychological impairment with higher performance among older versus younger people with HIV (*P*=.03). While the types of factors relating to cognition were expected [26,31], the relationships were not always in the anticipated direction. For example, our finding global neuropsychological impairment was higher in younger compared to older people with HIV is counterintuitive. At present, we are uncertain as to why this pattern was present. However, this finding is hypothesis generating and suggests the importance of cognitively screening people before the age of 50 years. It also remains unclear as to why people with HIV with Hispanic ethnicity were more likely to be impaired (*P*=.03). The *T* scores were demographically corrected for race/ethnicity as well as adjusted for age, sex, and education; and the tests in BRACE are not literacy dependent as both written and verbal instructions (via video) are provided.

The prevalence of cognitive impairment detected in outpatient clinical care suggests the need for HIV services that incorporate routine, brief cognitive screening into patient management for numerous reasons. Detection of cognitive impairment is necessary to adequately manage patient care and potentially improve clinical outcomes because, in its severe form, impairment may impact everyday functioning including attending routine HIV clinic care, financial and medication management, driving, multitasking, and vocational functioning [30,32-34]. Continued cognitive screening also allows for the ability for early detection, management, and intervention of mild forms of cognitive impairment that may either progress or fluctuate over time. As the mechanisms underlying cognitive impairment are likely complex and multifactorial, routine cognitive screening is necessary at minimum to determine whether modifiable factors (eg, medications with anticholinergic burden or polypharmacy for comorbid conditions [35], antiretroviral therapy medications such as efavirenz- [36] or dolutegravir-based regimens) can lead to impairment (although not seen in the present study) and thus remedied by the clinician. To accomplish routine cognitive screening, resources would need to be allocated to cognitive screening. For instance, iPads would be needed if BRACE were to be implemented in clinic. Clinicians would also need to be trained on the tool for examining the results and determining any subsequent recommendations. For instance, if individuals demonstrate impairment via cognitive screening, further neuropsychological evaluation by a trained professional (ie, neuropsychologist) should be recommended to better understand domain-specific impairment because there is significant heterogeneity in cognitive function in people with HIV [9,37,38]. Not all individuals demonstrate the same neuropsychological profile and different impairment profiles may result from different predictors or different mechanisms. Further evaluation is also necessary to determine whether impairment identified by BRACE may have been due to disinterest or poor engagement with testing or malingering for secondary gain (eg, disability).

There are a number of study limitations including the cross-sectional study design, which precludes any discussion of causality, as well as possible self-selection bias or lack of generalizability as participants voluntarily chose to enroll in this study. Our lack of an HIV seronegative, at-risk comparison group was also a major study drawback. An HIV-uninfected control group would have enabled the direct comparison of the prevalence of cognitive impairment using the BRACE in people with HIV seeking routine clinic care compared to an uninfected control group after adjusting for any relevant sociodemographic, behavioral, and clinical factors. As our primary interest was in the implementation of BRACE in the context of routine clinic HIV care and the prevalence estimates of cognitive impairment among these patients, we did not seek a control group. However, it is important that our cohort of people with HIV was standardized to an external group of HIV-uninfected individuals, which is standard practice in clinical neuropsychology. While our *T* scores were estimated using a normative based regression approach (adjusted for age, sex, race/ethnicity, and education; T Marcotte, unpublished data), follow-up scores were not adjusted for practice at this point as those regression equations are currently being developed. This is important as the lack of a practice effect in people with HIV may suggest impairment. Furthermore, it is also important to note that our *T* scores were estimated based on a sample of only 144 HIV-uninfected individuals aged 18 to 70 years. Our sample ranged in age from 21.6 to 79.3 years, with 5 people with HIV over the age of 70 years. Larger samples of HIV-uninfected individuals, particularly those individuals over 70 years of age, will be collected to refine these demographic adjustments. It may also be possible to better refine the cutpoints that maximize sensitivity and specificity for impairment, using more robust regression models. That work, in various cohorts, is underway. Another limitation was that our smaller sample of people with HIV completing a lengthier cognitive battery comprised fewer males than the larger sample. This study provides the groundwork for additional studies examining the psychometric properties of BRACE. Additionally, our measurement of clinical and behavioral comorbidities from ICD-10 is not optimal, particularly, for mental health (eg, depression or anxiety) and substance use disorders, which can fluctuate with management. Electronic medical record extraction of conditions is also not always comprehensive although it can be a rich data source. Future studies will be needed to look more deeply at better measurements of comorbidities in conjunction with BRACE. Additionally, at this point, we were unable to assess important covariates in this study including polypharmacy, which has shown to be associated with increased cognitive impairment [35]. Generalizability at this point is also limited to a predominately low educated, African-American, older people with HIV seeking outpatient clinic care, which is an important understudied population. Determining the clinical utility of BRACE in other US populations and internationally is warranted.

To continue to address cognitive impairment moving forward, traditional neuropsychological assessments are necessary but are often not conducted due to feasibility of available neuropsychologists as they typically have long wait lists. Thus, many persons with milder but clinically relevant cognitive impairment go undetected and without intervention. Sensitive and rapidly obtainable metrics that are obtained continuously, ubiquitously, and proactively in real time such as BRACE (an expanded cognitive screener) are needed. Other technology-based tools that differ from BRACE (eg, length of assessment; administrator-assisted; computer, tablet, or phone-based) have also been developed or used to screen for cognitive impairment in HIV including NeuroScreen [29,39], the Computer Assessment of Mild Cognitive Impairment [40], and CogState [41]. The primary advantage of BRACE is that it was designed to be self-administered versus administrator-assisted. Rapid advancement of iPad-based technologies have increased our ability to effectively screen cognitive impairment in busy clinics where HIV providers have limited time to manage patients with multimorbidity (eg, multiple medical, psychiatric, and cognitive conditions) and polypharmacy (eg, multiple antiretroviral therapy and nonantiretroviral therapy drugs in use). In addition, ubiquitous access to the internet enables updating of norms and the real-time calculations of risks. BRACE appears to provide the field with an effective user- and clinician-friendly cognitive screener that has the potential to influence patient care for identifying cognitive impairment (eg, identify a patient that may have been missed or identified too late), tracking performance over time, and determining prediction models of cognitive impairment. The results of BRACE can also inform the neuropsychological assessments which can expand upon the initial screen. While larger, longitudinal studies across heterogeneous subgroups of people with HIV and HIV-uninfected individuals in primary care are needed, our study provides initial evidence for the utility of this tool in predominately African-American older people with HIV with low levels of education seeking outpatient clinic care.

## Appendix

Multimedia Appendix 1 Supplementary tables.

## Abbreviations

AIDS: acquired immunodeficiency syndrome
BRACE: Brain Baseline Assessment of Cognition and Everyday Functioning
HIV: human immunodeficiency virus
ICD-10: International Statistical Classification of Disease, tenth revision
TMT: Trail Making Test
