# Supplementary material for: Tablet-Based Cognitive Impairment Screening for Adults With HIV Seeking Clinical Care: Observational Study
Source: JMIR Ment Health. 2021 Sep 9;8(9):e25660. doi: 10.2196/25660 (PMC8461534; doi:10.2196/25660)
Supplement: Multimedia Appendix 1 [file mental_v8i9e25660_app1.docx]

**Supplemental Table 1**. Comparison of sociodemographic and clinical factors between the subgroup of people with HIV (PWH) completing a gold standard neuropsychological (NP) test battery versus the larger group of PWH not completing the NP test battery.

|  | | NP data available  (n=61)  n (%) | No NP data available (n=343)  n (%) | *P*-value |
| --- | --- | --- | --- | --- |
| Age, *mean (standard deviation)* | | 55.9 (8.2) | 53.2 (11.0) | .06 |
| Male | | 14 (23) | 216 (63) | <.001 |
| Years of Education | |  |  | .19 |
|  | Less than high school | 24 (39) | 95 (28) |  |
|  | High school | 23 (38) | 149 (44) |  |
|  | More than high school | 14 (23) | 97 (28) |  |
| African-American/Black | | 55 (90) | 277 (81) | .08 |
| Hispanic/Latino | | 2 (3) | 15 (4) | .69 |
| Current CD4 count^a^ | |  |  | .29 |
|  | Less than 200 | 42 (70) | 222 (66) |  |
|  | 200-500 | 15 (25) | 76 (22) |  |
|  | More than 500 | 3 (5) | 40 (12) |  |
| Current HIV RNA (cp/ml) ^a^ | |  |  | .96 |
|  | Undetectable (<20) | 40 (66) | 228 (67) |  |
|  | Less than 200 | 13 (21) | 70 (21) |  |
|  | Greater than 200 | 8 (13) | 41 (12) |  |
| On ART | | 61 (100) | 341 (99) | .55 |
| On ART and undetectable HIV RNA | | 40 (65) | 227 (66) | .93 |

^a^4 participants missing data; antiretroviral therapy (ART) included are agents used by more than 5% of the sample. *P*-values statistically compare the distributions of covariates in those with NP data available and those without.

**Supplemental Table 2**. Performance on the standard neuropsychological test battery among 61 people with HIV.

|  | | M (SD) | Range |
| --- | --- | --- | --- |
| SDMT | | -0.27 (1.26) | -3.04 – 2.31 |
| Trail Making Test | |  |  |
|  | Part A | -0.55 (2.25) | -8.75 – 3.89 |
|  | Part B | -0.99 (2.63) | -10.06 – 3.38 |
| Semantic fluency | | 0.05 (1.01) | -1.80 – 2.04 |
| HVLT-R | |  |  |
|  | Total learning | -1.00 (1.14) | -3.34 – 1.43 |
|  | Delay free recall | -1.61 (1.63) | -4.68 – 1.82 |
| Grooved Pegboard | |  |  |
|  | Dominant | -1.72 (3.12) | -17.43 – 1.77 |
|  | Nondominant | -1.88 (3.54) | -16.23 – 2.07 |

Note. M=mean; SD=standard deviation; SDMT=Symbol Digit Modalities Test

**Supplemental Table 3**. Performance from 404 PWH on the BRACE.

|  | M (SD) | Range |
| --- | --- | --- |
| TMT-Part A | 44.98 (10.74) | 15.85 – 73.04 |
| TMT-Part B | 42.37 (9.29) | 9.22 – 70.71 |
| Stroop-Test | 43.23 (10.54) | 11.27 – 74.62 |
| VSLT | 47.71 (8.43) | 21.63 – 79.82 |

Note. TMT=Trail Making Test; VSLT=Visual Spatial Learning Test.
